# High-Speed Video Microscopy for Primary Ciliary Dyskinesia Diagnosis: A Study of Ciliary Motility Variations with Time and Temperature

**DOI:** 10.3390/diagnostics11071301

**Published:** 2021-07-20

**Authors:** Ana Reula, Javier Pitarch-Fabregat, Javier Milara, Julio Cortijo, Manuel Mata-Roig, Lara Milian, Miguel Armengot

**Affiliations:** 1 Pathology Department, University of Valencia, 46010 Valencia, Spain; manuel.mata@uv.es (M.M.-R.); lara.milian@uv.es (L.M.); 2 Biomedical Sciences Department, Cardenal Herrera-CEU University, 12006 Castellón, Spain; 3 Molecular, Cellular, and Genomic Biomedicine Group, IIS La Fe, 46020 Valencia, Spain; miguel.armengot@uv.es; 4 Internal Medicine Service, Consortium of Hospital General Universitario de Valencia, 46014 Valencia, Spain; javierpitf@hotmail.com; 5 Pharmacy Service, Hospital General Universitario de Valencia, 46014 Valencia, Spain; xmilara@gmail.com; 6 Pharmacology Department, Jaume I University, 12006 Castellón, Spain; 7 CIBER of Respiratory Diseases (CIBERES), Carlos III Health Institute, Ministerio de Ciencia e Innovación, 28029 Madrid, Spain; 8 Pharmacology Department, University of Valencia, 46010 Valencia, Spain; julio.cortijo@uv.es; 9 Teaching and Research Unit, Consortium of Hospital General Universitario de Valencia, 46014 Valencia, Spain; 10 ENT Service, La Fe Politechnic and University Hospital, 46020 Valencia, Spain; 11 Surgery Department, University of Valencia, 46010 Valencia, Spain

**Keywords:** primary ciliary dyskinesia (PCD), ciliary beat frequency (CBF), ciliary beat pattern (CBP), high-speed video microscopy (HSVM)

## Abstract

Primary ciliary dyskinesia (PCD) is a rare disease resulting from a defect in ciliary function that generates, among other issues, chronic upper and lower respiratory tract infections. European guidelines recommend studying ciliary function (pattern (CBP) and frequency (CBF)), together with characteristic clinical symptoms, as one of the definitive tests. However, there is no “gold standard”. The present study aims to use high-speed video microscopy to describe how CBF and CBP alter over time and at different temperatures to reduce the error rate in the diagnosis of PCD. Samples of nasal epithelium from 27 healthy volunteers were studied to assess CBF and CBP at 0, 3, 24, 48, and 72 h, at room temperature and 4 °C. It was observed that CBF increased while CBP became dyskinetic, both at room temperature and at 4 °C, as time passed, especially after 3 h. In order to preserve all ciliary function parameters and to perform a reliable analysis to improve the diagnostic process of PCD, analysis should be performed within the first 3 h of sample collection, preferably in reference centers.

## 1. Introduction

Primary ciliary dyskinesia (PCD) is a rare disease with a prevalence of 1:20,000 live births (OMIM 242650, ORPHA244). It is a genetic disease characterized by an alteration in the motility pattern and/or frequency of motile cilia and flagella. This results in a deficit in mucociliary clearance, causing infections and chronic inflammation of the airways. This disease is also associated with fertility problems, and the distribution of organs in these patients is random, resulting in situs inversus in 50% of cases (known as Kartagener’s syndrome) and situs ambiguous in 6% [1].

At present, there is no “gold standard” technique for the diagnosis of PCD [2], and a combination of different tests is required: analysis of the ciliary beat pattern (CBP) and ciliary beat frequency (CBF) [3] by high-speed video microscopy (HSVM) [4,5], the study of ciliary ultrastructure by transmission electron microscopy (TEM) [6], and genetic diagnosis [7]. In addition, nasal nitric oxide (nNO) determination is sometimes used as a screening test [8], and immunofluorescent labeling of specific proteins of the ciliary structure is emerging as another potential test that still needs to be validated [9]. Although TEM and genetics are two definitive diagnostic tests, 20–30% of cases of PCD show [9] normal ciliary ultrastructure. PCD is a multigenic disorder, as evidenced by the identification of more than 40 genes related with the diseases; these, however, are only present in 60–70% of the studied families. These circumstances imply a relatively high false-negative rate in PCD diagnosis. Therefore, ciliary function analysis by HSVM is still considered a key test, according to the European Guidelines for the diagnosis of PCD [2,10,11,12].

It has been demonstrated [13] that air–liquid interface culture of PCD epithelial cells before analyzing ciliary function and ultrastructure is a robust and informative diagnostic aid that helps to reduce false positives due to secondary defects. However, there are important disadvantages when comparing with direct observation of the samples of nasal ciliary cells: (1) ALI culture requires highly specialized personnel and expensive material; (2) the success rate of ALI-cultured nasal epithelium varies from 54% to 79%. Since the diagnostic process is costly and time-consuming, and requires specialized and trained staff, it is performed in reference centers [8]. One of the main problems of this rare disease is that it is often diagnosed late, worsening prognostics, especially at the respiratory level [14]. The improvement of diagnostic tests’ effectiveness is necessary for an early PCD diagnosis. HSVM is a key test that must be carried out in reference centers, which are often located far from patients’ homes. It is necessary to know how time and the temperature at which the ciliated epithelium samples are transported to these centers affect the test results.

Therefore, in this study, our objectives were: (1) to describe how ciliary function parameters (CBP and CBF) vary in function of time and temperature conservation using HSVM from fresh samples of healthy volunteers; and (2) to determine what is the maximum time after sample collection at which assessment of ciliary function using HSVM could be carried out in order to reduce the error rate in the diagnosis of PCD using this test. The novelty of this work resides in the fact that, on the one hand, we provide information on the effect of temperature on CBP in relation to CBF and, on the other hand, we establish the time limits in which a sample can be reliably analyzed in a specialized unit for the diagnosis of PCD.

## 2. Materials and Methods

All epithelial cell samples were collected after informed consent was signed by the participants. The study protocol complied with the ethical criteria of the 1975 Declaration of Helsinki [15].

The study involved 27 healthy volunteers (age 27, 53 ± 8, 91) from whom samples of airway epithelial cells were obtained by curettage of the nasal middle turbinate mucosa. No local anesthetic was used to obtain the samples in order to avoid the possibility of interaction between the drug and the subsequent analysis of the ciliary movement. None of the volunteers were receiving nasal topical treatments, and none of them suffered from acute or chronic airway disease. Additionally, none of them were smokers.

Obtained tissue samples were disaggregated and dissolved (for transport and analysis) in 1 mL of Dulbeccois Modified Enaglesis Medium (DMEM, Cambrex, East Rutherford, New Jersey) supplemented with 10% fetal bovine serum, 100 U/mL of penicillin, 100 µg/mL of streptomycin, and 2 mM of glutamine.

CBP and CBF were determined in all samples. Prior to ciliary function analysis, each sample was divided in two to determine temperature-dependent differences. Half of the samples were kept at room temperature (22–24 °C) and the other half in a refrigerator (4 °C). For each sample, videos were recorded at 0, 3, 24, and 48 h after collection; only three of the samples were also analyzed at 72 h.

**Figure 1:**
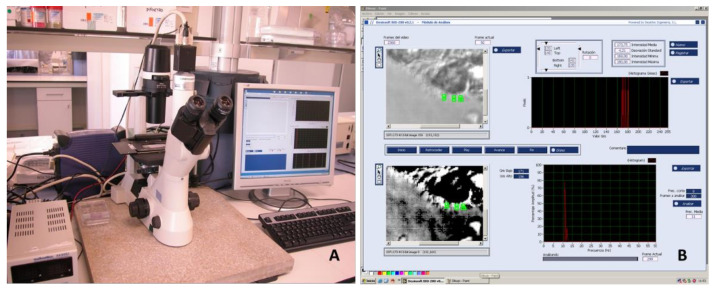
(**A**) Image of the microscope, camera and (**B**) high-speed digital video-imaging software developed for the analysis using Desinsoft-Bio 200 software (Valencia, Spain).

Visualization of the samples was performed at room temperature with the high-speed digital video-imaging system associated with a Nikon Eclipse TS100 microscope (Nikon, Tokyo, Japan). A phase-contrast objective with 40X magnification was used (Figure 1). A Multimetrix^®^ XA3051 (Dewsintec SL, Valencia, Spain) was used for on-line CBF measurement. Transillumination was performed with a 150 W green light filter, whose beam was directly connected to a CCD camera (CV-A33 CL Digital Quad High-Speed Progressive Scan Camera, Jai UK Ltd., Unbridge, UK), with a resolution of 649 × 494 pixels and a maximum resolution speed of 128 fps. Video signals were digitized and processed using Desinsoft-Bio 200 software (Valencia, Spain). The video images were analyzed by monitoring the variation in light intensity of each pixel using Fourier transform [16]. All statistical analyses were conducted using R software version 4.0, brms package version 2.12, and clickR package version 0.4.39.

For CBF determination, 3 area measurements were obtained in which a good ciliary image was captured in each condition. Any measurement below 7 Hz was not taken into account because it did not correspond to normal expected measurements, and it could be due to artefactual values due to methodological or biological reasons. Similarly, any measurement with an amplitude percentage higher than 40% was discarded because it was considered an excessively heterogeneous determination.

**Figure 2:**
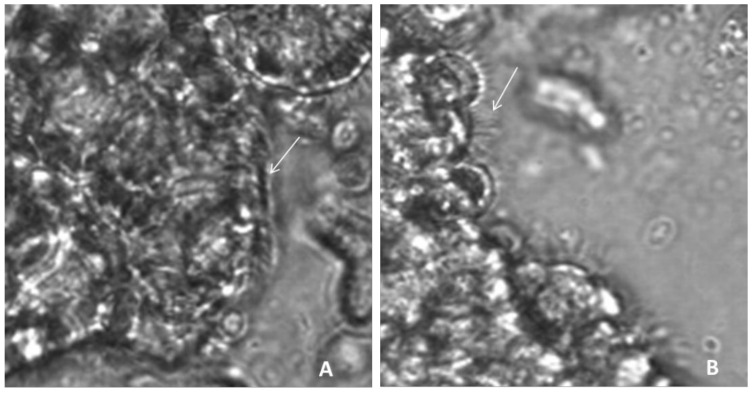
Samples of nasal ciliated epithelium: these images correspond to a frame of a high-speed video file (**A**). Example of cilia with normal function (arrow), consisting in movement of all phases with adequate amplitude and coordination. (**B**). Example of dyskinetic cilia area (arrow), with straight cilia, characteristic of vibratile movement.

The determination of CBP was made by direct visualization by an observer and was classified into two categories based on a modification of Chilvers MA et al. [17], considering that the beat pattern could be normal (N)—the ciliary beating consists of all phases with adequate amplitude and coordination—or dyskinetic, which would include the categories of vibratile (V)—the beating amplitude is decreased, and the cilia appear stiff—and uncoordinated (I), i.e., the cilia do not beat in synchronized metachronous phases (Figure 2). In order to evaluate CBP, cilia from at least 10 edges were examined. Cilia movement was registered for 1 min at 118 fps. The measurements were performed by two investigators using homogeneous criteria previously validated by a k-test. (>85%).

Data were summarized using mean (sd) and median (1st and 3rd quartiles) for numeric variables and absolute frequency (%) for qualitative variables. A Bayesian Gamma regression model was used to evaluate the evolution over time and its differences between the temperature on CBF values. Additionally, a Bayesian logistic regression model was used to assess the evolution over time and the differences between the temperature on dyskinetic CBP probability. These models considered a 2-way interaction between time and temperature. Given the longitudinal nature of the study and the non-independence of the observations, a slope for time and an independent term for each sample were included in the models. Furthermore, as the effect of CBP overtime was not expected to be linear, time was included in the model as a monotonic effect. Since the use of interactions made estimated coefficients difficult to interpret, focus was given to the partial dependence plots with their 95% confidence intervals and post hoc density probabilities.

## 3. Results

### 3.1. Ciliary Beat Frequency

**Figure 3:**
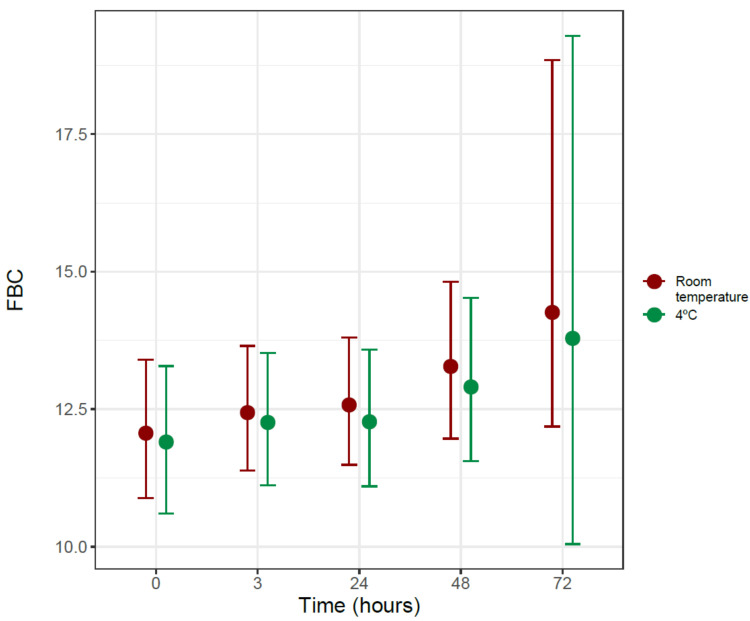
Ciliary beat frequency (CBF) in Hz as a function of time at room temperature (22–24 °C) and at refrigerator temperature (4 °C).

Table 1 shows the ciliary beat frequency measurements, expressed in Hz using the mean and standard deviation, at the different times and temperatures. Figure 3 shows the evolution of CBF as a function of time, both at room temperature and 4 °C, from the Bayesian Gamma regression model performed.

The Bayesian Gamma regression model shows that time is associated with an increase in the ciliary beat frequency, with a probability (PD) of 97.51% and a confidence interval very close to statistical significance (Exp (estimate) 1.047 CI95% [0.999; 1.121]). When comparing the data obtained at room temperature with those obtained in the refrigerator, no differences were observed between them, neither at time 0 (Exp (estimate) 0.986 CI95% [0.903; 1.072]) nor when comparing their evolution over time (Exp (estimate) 0.992 CI95% [0.908; 1.069]).

### 3.2. Ciliary Beat Pattern

Table 2 shows the absolute and relative frequencies of the normal and dyskinetic ciliary beat pattern, including vibratile and uncoordinate, as well as the proportions at each time and temperature.

**Figure 4:**
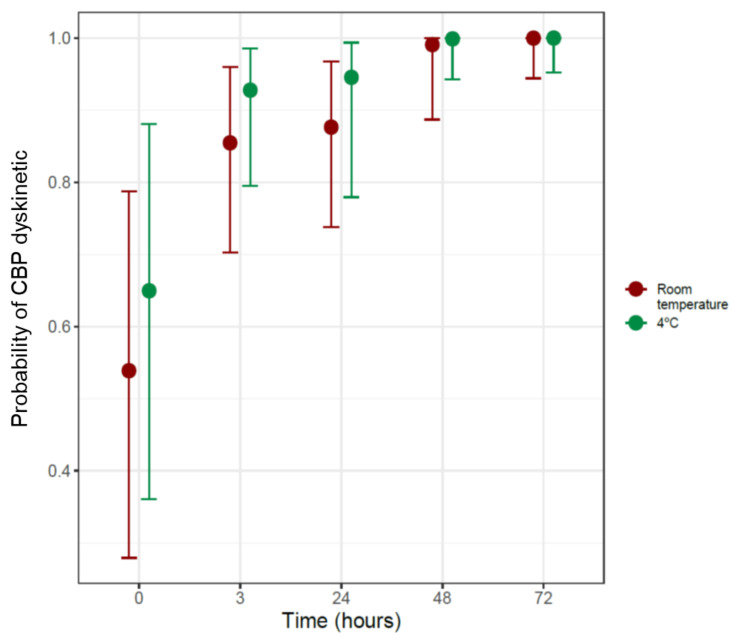
Probability of dyskinetic ciliary beat pattern as a function of time at room temperature and 4 °C.

A Bayesian logistic regression model was performed in this case, represented in Figure 4, showing the probability of the appearance of a dyskinetic ciliary beat pattern (vibratile + uncoordinated) at room temperature and at 4 °C as a function of time.

It was observed how, at room temperature, as a function of time, the probability of the ciliary beat pattern being dyskinetic increased significantly (OR = 33, 95%CI [1.79, 1165]), with a probability greater than 99%. When comparing these data with those observed in samples preserved at 4 °C, no significantly differences were observed (OR = 2.59, 95%CI 0.2, 352.5), so it can be deduced that the probability of presenting a dyskinetic pattern at 4 °C also increases as a function of time. No differences were observed when comparing the samples at baseline to time 0 (OR = 1.6, 95%CI 0.41, 7.15).

## 4. Discussion

In the present work, we have studied the variation in CBF and CBP at different temperatures and conservation times in nasal epithelial biopsies from healthy individuals, with the aim of defining a protocol to improve the efficacy of HSVM as a diagnostic test for PCD.

A similar study was performed by Sommer et al. in 2010, but only focused on the change in CBF [18]. As previously stated, to assess ciliary function, CBP (and not CBF) is the main parameter to evaluate when studying a case of PCD by HSVM, since there are phenotypes with normal CBF in which only CBP is altered [17,19,20]. According to the European Respiratory Guidelines, CBP, together with a consistent clinical history, may be more useful to diagnose these patients than ciliary ultrastructure analysis by transmission electron microscopy, as in many cases it can be normal [21,22]. The results show how, over time, there are increases in CBF in nasal epithelial samples. Despite not being clearly defined in the bibliography, this increase could be due to the mechanism of ciliary movement regulation carried out by dynein proteins, which are regulated by cAMP-controlled phosphorylation.

Among other factors, intracellular calcium increases CBF, first through a mechanism independent of PKA and then through mechaninsms dependent on cAMP increases [23,24,25]. Regarding the increase in CBF at 72 h, it cannot be considered as a strong evidence, because it could be due to the low sample size.

The evaluation of the ciliary beat pattern is fundamental for the diagnosis of PCD. In this regard, it should be noted that, in our work, CBP changes over time in a significant way, as a function of time, in favor of dyskinetic patterns, showing a rapid decrease in patterns considered normal.

The literature shows controversy regarding the preservation of samples at 4 °C [20]. In our study, there is a decreasing trend in normal CBP in favor of vibratile and uncoordinated CBP at this temperature. Alterations in CBP are due to problems in cilia beating coordination and amplitude. However, there were areas with cilia beating at normal frequencies, despite the low temperature. We believe that preservation at room temperature introduces fewer external factors that can alter the analysis, and thus could provide advantages over low temperature preservation. Although some centers make measurements using a heating plate at 37 °C, in others, such as our own, the analysis of samples is performed at room temperature, since the aim of the work is to determine how the sample preservation temperature affects CBP rather than to ascertain the temperature at which the measurement is made.

Few centers have both the technical and human resources to perform ciliary beat pattern assessments. The alteration in CBP during the first hours after sample collection must be taken into account. As previously mentioned, the PCD diagnostic pathway is complex and expensive. A patient’s displacement to the reference center to obtain the sample and its analysis within the first 3 h would significantly decrease the rate of false positives and non-assessable samples, thus improving the diagnostics of PCD.

Despite the low number of samples analyzed, our results indicate a clear trend in the variation of ciliary beat frequency as a function of time, as well as statistically significant differences in the variation of ciliary beat patterns as a function of time.

## 5. Conclusions

Ciliary beat frequency increase in a time-dependent manner due to alterations in CBP.

The ciliary beat pattern is one of the most valuable tools for the diagnosis of primary ciliary dyskinesia. It is, however, markedly altered depending on the time after sample extraction, with dyskinetic patterns appearing both at room temperature and at 4 °C, especially after 3 h.

The analysis of nasal epithelium samples from patients suspected of having primary ciliary dyskinesia should be performed early, preferably within the first 3 h after collection, and kept at room temperature. This analysis should be carried out in reference centers in order to guarantee the correct processing of samples and therefore the correct diagnosis of PCD.

## Figures and Tables

**Table 1:** Ciliary beat frequency (CBF) in samples of healthy subjects measured under different conditions, expressed as mean (Hz) and standard deviation.

	Room T (22–24 °C)	Refrigerator T (4 °C)
0 h	11.99 (3.34)	10.79 (1.80)
3 h	10.86 (2.19)	12.08 (2.46)
24 h	12.56 (3.03)	11.98 (2.24)
48 h	12.74 (3.21)	12.53 (3.54)
72 h	17.78 (1.80)	14.03 (4.51)

**Table 2:** Frequencies of ciliary beat patterns as a function of time and temperature.

	Room Temperature (22–24 °C)			Refrigrator Temperature (4 °C)		
	Normal	Dyskinetic		Normal	Dyskinetic	
	Normal	Uncoordinated	Vibratile	Normal	Uncoordinated	Vibratile
**0 h**	9 (52.9%)	1 (5.6%)	7 (41.2%)	6 (42.9%)	3 (21.4%)	5 (35.7%)
**3 h**	7 (43.6%)	4 (25.0%)	5 (31.3%)	2 (20.0%)	2 (20.0%)	6 (60.0%)
**24 h**	0 (0.0%)	9 (40.9%)	13 (59.1%)	1 (6.2%)	8 (50.0%)	7 (43.8%)
**48 h**	1 (5.3%)	4 (21.0%)	14 (73.7%)	0 (0.0%)	5 (33.3%)	10 (66.7%)
**72 h**	0 (0.0%)	1 (33.3%)	2 (66.7%)	-	-	3 (100%)
